# Biomarkers of Hypercoagulability in COVID-19

**DOI:** 10.3390/jcm12103525

**Published:** 2023-05-17

**Authors:** Hideo Wada

**Affiliations:** Mie Prefectural General Medical Center, Department of General and Laboratory Medicine, Yokkaichi 510-8561, Japan; wadahide@clin.medic.mie-u.ac.jp; Tel.: +81-59-345-2321

This issue focuses on the pathophysiology of coronavirus disease 2019 (COVID-19) [1,2,3,4,5,6] and several biomarkers for hypercoagulability in patients with disseminated intravascular coagulation (DIC) [7,8,9], acute cerebral infarction (ACI) [7,10], acute myocardial infarction (AMI) [7], venous thromboembolism (VTE) [7], and COVID-19 [5,6]. Patients with COVID-19 develop hypercoagulability, and COVID-19 is frequently associated with various thromboses. Soluble C-type lectin-like receptor 2 (sCLEC-2) is released from activated platelets [11], and fibrinogen and fibrin degradation products (FDPs), D-dimer, and soluble fibrin (SF) include fibrin-related markers (FRMs) [7]. Thus, sCLEC-2 is considered to be a biomarker for platelet activation, as well as microvascular or arterial thrombi, and FRMs are considered to be biomarkers for venous and microvascular thrombi. Although elevated levels of these biomarkers suggest hypercoagulability in various pathological states, the mechanisms underlying the elevation of these biomarkers differ between diseases. In particular, sCLEC-2 is a new biomarker for platelet activation that has not been routinely established for clinical use, suggesting that further evidence will be needed prior to its use. Both FDP and D-dimer, which are used in the scoring system for the diagnosis of DIC, are the best biomarkers for diagnosing DIC. They are also useful for the exclusion of VTE, but not for the diagnosis of AMI or ACI [7,8]; on the other hand, SF is useful for the diagnosis of not only DIC and VTE, but also AMI and ACI. Although elevated sCLEC-2 levels are considered useful for the diagnosis of DIC, AMI, and ACI, in which platelet activation occurs, sufficient evidence for the diagnosis of thrombosis has not yet been established (Figure 1). As markedly high sCLEC-2 levels are observed in patients with COVID-19, platelet activation has been considered to occur in patients with COVID-19. However, while COVID-19 is frequently complicated by VTE, it is not affected by AMI or ACI, suggesting that multiple mechanisms for the onset of thrombosis might exist in COVID-19 [12].

## Conclusions

These biomarkers are generally useful for the diagnosis of each type of thrombosis, and might be useful for the prediction of thrombotic state in patients with COVID-19.

## Figures and Tables

**Figure 1:**
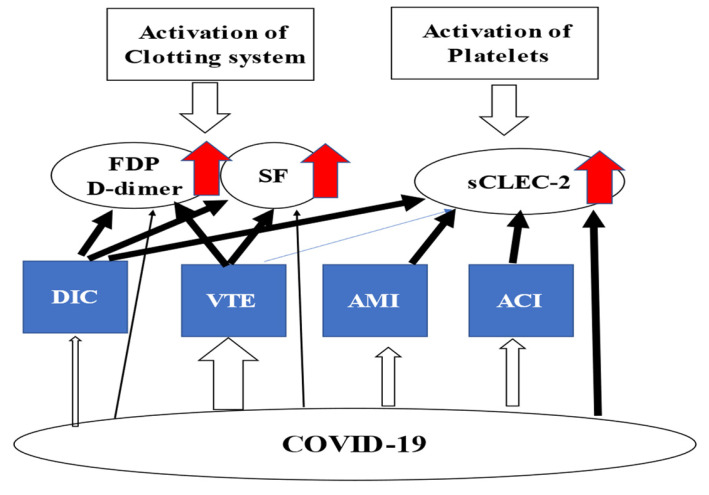
Biomarkers for hypercoagulability. COVID-19—coronavirus disease 2019; DIC—disseminated intravascular coagulation; ACI—acute cerebral infarction; AMI—acute myocardial infarction, VTE—venous thromboembolism; sCLEC-2—Soluble C-type lectin-like receptor 2; FDP—fibrinogen and fibrin degradation products; SF—D-dimer and soluble fibrin.
